# Chronic Thrombosed Abdominal Aortic Aneurysm Treated by Endovascular Aneurysm Repair Using Bifurcated Endograft: A Case Report

**DOI:** 10.70352/scrj.cr.26-0045

**Published:** 2026-05-20

**Authors:** Mitsuru Nakanishi, Koichi Morisaki, Shinichiro Yoshino, Daichi Ito, Kohei Ueno, Yusuke Fujioka, Go Kinoshita, Kentaro Inoue, Tomoharu Yoshizumi

**Affiliations:** Department of Surgery and Science, Graduate School of Medical Sciences, Kyushu University, Fukuoka, Fukuoka, Japan

**Keywords:** chronic thrombosed abdominal aortic aneurysm, endovascular aneurysm repair, bifurcated stent graft

## Abstract

**INTRODUCTION:**

Thrombosed abdominal aortic aneurysms (AAAs) are rare. Owing to technical difficulties and concerns regarding inadequate device deployment, open surgical repair (OSR) has traditionally been performed rather than endovascular aneurysm repair (EVAR).

**CASE PRESENTATION:**

A 70-year-old male presented with bilateral intermittent claudication caused by a chronically thrombosed AAA, 53 mm in diameter. Although the occlusion extended from the AAA to the bilateral external iliac arteries, EVAR was planned based on the patient’s clinical background. The occluded lesion was crossed using a bidirectional approach, and EVAR was successfully performed using a bifurcated stent graft.

**CONCLUSIONS:**

This case report demonstrates that EVAR for a chronically thrombosed AAA can be performed by applying endovascular techniques commonly used to treat lower-extremity arterial disease.

## Abbreviations

AAA
: abdominal aortic aneurysm
ABI
: ankle–brachial index
ACT
: activated clotting time
ALI
: acute limb ischemia
AUI
: aorto-uni-iliac
BA
: brachial artery
CFA
: common femoral artery
CIA
: common iliac artery
EIA
: external iliac artery
EVAR
: endovascular aneurysm repair
IFU
: instructions for use
IVUS
: intravascular ultrasonography
LEAD
: lower extremity arterial disease
OSR
: open surgical repair
TEVAR
: thoracic endovascular aortic repair

## INTRODUCTION

Thrombosed AAAs are relatively rare, occurring in 0.6%–2.8% of AAAs.^1–3)^ Acute occlusion of a thrombosed AAA results in ALI,^4)^ whereas chronic occlusion of a thrombosed AAA typically presents with intermittent claudication of the lower extremity.^5)^ Although the AAA sac is generally presumed to be filled with a thrombus, sac enlargement and subsequent AAA rupture have been described even in thrombosed AAAs.^6)^ EVAR has low early morbidity and mortality, and has become a widespread alternative and less invasive treatment option than OSR.^7,8)^ EVAR has been increasingly used in patients previously treated with OSR.^9)^ However, because of the technical difficulties in advancing a guidewire through an occluded AAA and concerns regarding inadequate device deployment,^10,11)^ OSR has traditionally been performed instead of EVAR for the treatment of thrombosed AAAs.^12)^ With recent advances in endovascular techniques and devices for LEAD, especially in the aortoiliac area, thrombosed AAAs have been treated with EVAR.^4,5,10,11,13–15)^ However, most reported cases involved emergent/urgent EVAR for acute thrombotic occlusion,^4,10,11,13–15)^ and elective EVAR for chronically thrombosed AAA has not been well reported. Karkos et al.^5)^ reported elective EVAR for a chronically thrombosed AAA using an AUI device. Herein, we present a technically successful case of elective EVAR using a bifurcated endograft for a thrombosed AAA.

## CASE PRESENTATION

**Fig. 1:**
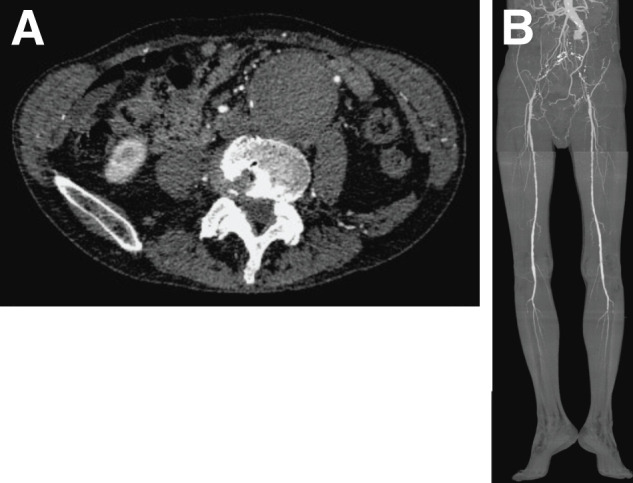
Preoperative CTA images. (**A**) A 53-mm AAA with complete thrombosis of the aneurysm lumen. (**B**) 3D maximum intensity projection image. AAA, abdominal aortic aneurysm

A 70-year-old male presented with bilateral intermittent claudication. On physical examination, femoral and distal pulses were absent bilaterally. The ABI was 0.38 on the right leg and unmeasurable on the left leg. Preoperative CTA revealed an infrarenal thrombosed AAA, 53 mm in diameter (**Fig. 1A**). The proximal aortic neck superior to the AAA was patent. Although the proximal aneurysm sac had a slit-like residual lumen, the sac was occluded by a thrombus. The occlusion extended bilaterally to the EIAs. The bilateral CFAs and distal vessels remained patent through the collateral circulation (**Fig. 1B**). Partial calcification was observed in the aneurysm wall and bilateral CIAs. The inferior mesenteric artery was patent and originated from the junction of the proximal neck and the aneurysmal sac. The infrarenal neck had a diameter of 23 mm and a length of 45 mm. The right CIA had a diameter of 20 mm and a length of 40 mm, whereas the left CIA had a diameter of 25 mm and a length of 35 mm. The diameters of the right and left EIAs were 7.9 and 8.0 mm, respectively. In addition, a 4-cm mass was identified adjacent to the descending thoracic aorta, and lung cancer was diagnosed after further evaluation.

We decided to perform surgical intervention based on the following considerations: (1) presence of intermittent claudication, (2) persistent risk of rupture even in thrombosed AAAs, and (3) potential need for concomitant TEVAR as a prophylactic measure against vascular injury during surgical resection of lung cancer adjacent to the thoracic aorta. To facilitate early transition to lung cancer treatment, EVAR was selected instead of OSR, given its low invasiveness and faster postoperative recovery.^16,17)^

**Fig. 2:**
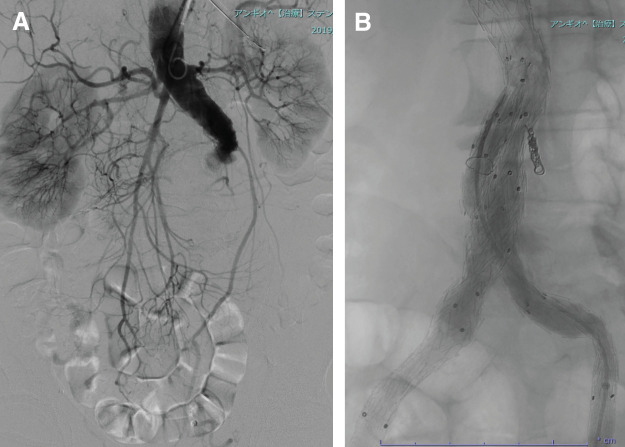
(**A**) Intraoperative aortography showing complete occlusion of the AAA and a patent proximal aortic neck. (**B**) Completion aortography during EVAR. AAA, abdominal aortic aneurysm; EVAR, endovascular aneurysm repair

Under general anesthesia, the patient was placed in the supine position. Oblique skin incisions were made on both groins. The bilateral CFAs were exposed, and 11-cm, 6-Fr sheath (Terumo, Tokyo, Japan) were inserted. The left BA was percutaneously punctured using ultrasonography, and a 90-cm, 5-Fr guiding sheath (Terumo) was inserted. Systemic anticoagulation was initiated with an intravenous bolus of 3000 units of heparin at the start of the procedure, with additional doses administered to maintain an ACT ≥200 s. The initial intraoperative aortography is shown (**Fig. 2A**). The chronic total occlusion extending from the thrombosed AAA to the right EIA was successfully crossed using a bilateral approach via the left BA and right CFA. A 180-cm, 0.018-inch, 12-gf guidewire (Asahi Intecc, Aichi, Japan) under the support of a 135-cm, 1.8/2.6-Fr microcatheter (Cook Medical, Bloomington, IN, USA) and a 120-cm, 4-Fr straight catheter (Tokai Medical Products, Aichi, Japan) was used for the antegrade approach. A 0.018-inch, 180-cm 12-gf guidewire (Asahi Intecc) supported by a 90-cm, 2.6-Fr microcatheter (Cook Medical) and a 65-cm, 5-Fr vertebral catheter (Cook Medical) were used for the retrograde approach. Subsequently, a pull-through wire was placed in the occluded right EIA. After confirmation of true lumen passage using IVUS, the lesion was dilated with a 4-mm diameter, 15-cm length balloon catheter (SABER; Cordis, Miami Lakes, FL, USA), followed by a 6-mm diameter, 10-cm length balloon (SABER; Cordis). After crossing the left side in the same manner, the Endurant II main body (28 × 16 × 145 mm; Medtronic, Minneapolis, MN, USA) was inserted via the right CFA. The main body was deployed using the push-up^18)^ and reverse slider technique,^19)^ and the contralateral gate in the flow-divider zone opened smoothly. The contralateral gate was cannulated using a 0.035-inch diameter, 260-cm length guidewire with the support of a 65-cm, 5-Fr vertebral catheter. A covered stent (10 mm × 10 cm) was deployed in the left EIA; the sheath was exchanged for a 33-cm, 14-Fr sheath (Medtronic), and the Endurant iliac limb (16 × 13 × 156 mm) was deployed from the contralateral gate to the left EIA. After the complete deployment of the ipsilateral leg of the main body, a covered stent (11 × 10 cm) was placed in the right EIA. The sheath was replaced with a 33-cm, 16-Fr sheath (Medtronic), and the Endurant iliac limb (16 × 13 × 124 mm) was deployed from the ipsilateral leg to the right EIA. Moderate compression was observed in the bilateral leg stent grafts, and additional stents were deployed in the inner lining to reinforce the existing stent grafts. Completion angiography revealed a good blood flow through the implanted endografts and stents (**Fig. 2B**).

**Fig. 3:**
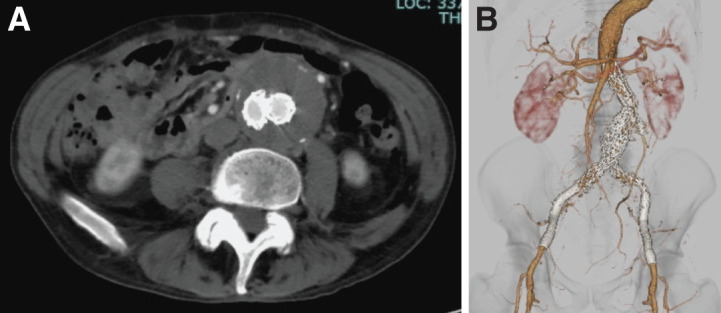
CTA images 4 days after EVAR. (**A**) Axial view of the aneurysm. (**B**) 3D virtual reality image. EVAR, endovascular aneurysm repair

Postoperatively, continuous intravenous heparin (10000 units/day) was continued until the next morning, and aspirin (100 mg/day) was initiated. The bilateral intermittent claudication improved. The ABI increased to 1.06 on the right and 0.98 on the left, and postoperative CTA showed good patency of the implanted stent grafts and no endoleak (**Fig. 3A** and **3B**). The patient was discharged without perioperative complications. During postoperative surveillance, brain metastasis from the lung cancer was diagnosed after EVAR, and surgery for the lung cancer was not performed. Unfortunately, the patient died 6 months after EVAR due to lung cancer. The EVAR endografts remained patent during surveillance.

## DISCUSSION

In the present case, elective EVAR with a bifurcated endograft was successfully performed for a thrombosed AAA using endovascular techniques commonly used in the treatment of LEAD. In this case, the lesion was considered a chronic occlusion based on the history of intermittent claudication for several years without ALI, the presence of well-developed collateral circulation, and CTA findings suggestive of an organized thrombus. Seven cases treated with EVAR were reported between 2005 and 2025 (**Table 1**). Of these, 6 cases involved acute occlusion of an AAA presenting with ALI; EVAR for a chronically thrombosed AAA has been reported in only 1 case, which used an AUI endograft. Terai et al.^10)^ and Uotani et al.^11)^ indicated that lesion crossing and device delivery are technically difficult when performing EVAR for thrombosed AAAs. Given the technical complexity of EVAR for thrombosed AAAs, some authors advocate OSR as the first-line treatment for thrombosed AAA.^2)^ OSR may be preferable in cases where lesion crossing and device delivery are difficult due to occlusive disease, in patients with proximal neck anatomy outside the IFU,^20)^ and in patients who can tolerate OSR. In the present case, the proximal neck anatomy was within the IFU, and EVAR was selected to allow for early treatment of advanced lung cancer. Even when EVAR is considered, an AUI approach is a reasonable option when lesion crossing is difficult or when a large thrombus burden may compromise contralateral gate deployment. In this case, an AUI approach was prepared as a backup strategy. Bilateral iliac lesion crossing was eventually achieved, allowing the successful deployment of a bifurcated stent graft.

**Table 1:** Reported cases of thrombosed AAA treated with EVAR

Author	Year	Age/sex	AAA size (mm)	Acute/chronic	Thrombosed area	Procedure	Device	Device configuration	Approach	Protection technique of distal embolism	Postoperative follow-up period (months)
Kumar^13)^	2005	58/M	38	Acute	AAA	EVAR	AneuRx	Straight	Bilateral femoral and left brachial	Aortic occlusion balloon and femoral clamping	10
Pillai et al.^14)^	2015	75/F	35	Acute	AAA	EVAR	Fluency	Straight	Left brachial	—	12
Terai et al.^10)^	2015	89/M	50	Acute	AAA	EVAR	Excluder	Bifurcated	Bilateral femoral	Femoral clamping	—
Uotani et al.^11)^	2018	77/M	42	Acute	AAA and bilateral EIA/IIA	Thrombectomy and EVAR	Excluder	Bifurcated	Bilateral femoral	Femoral clamping	12
Karkos et al.^5)^	2020	74/M	67	Chronic	AAA and bilateral CIA	EVAR	Endurant	AUI	Left axillary and left femoral	—	6
Robaldo et al.^15)^	2022	89/M	80	Acute	AAA and bilateral CIA	EVAR and femorofemoral bypass	Treovance	AUI	Right femoral	Femoral clamping	3
Xu et al.^4)^	2025	68/M	45	Acute	AAA and bilateral CIA	Thrombectomy and EVAR	Excluder	Bifurcated	Bilateral femoral	Femoral clamping	24

AneuRx and Enurant (Medtronic, Minneapolis, MN, USA); Excluder (W. L. Gore & Associates, Flagstaff, AZ, USA); Fluency (Bard Peripheral Vascular Inc., Tempe, AZ, USA); Treovance (Terumo Aortic [formerly Bolton Medical], Sunrise, FL, USA).

AAA, abdominal aortic aneurysm; AUI, aorto-uni-iliac; CIA, common iliac artery; EIA, external iliac artery; EVAR, endovascular aneurysm repair; F, female; IIA, internal iliac artery; M, male

Several technical aspects should be considered while performing EVAR for thrombosed AAAs. First, lesion crossing is essential but can be difficult due to the long occlusion length, especially in chronically occluded cases with an organized thrombus and hard fibrous plaques. In the present case, a bidirectional approach consisting of an antegrade approach through the left BA and a retrograde approach through the CFA was used. This approach, supported by IVUS, enabled successful and safe crossover of the occluded lesion. Second, there is a concern regarding insufficient expansion of the stent-graft leg within the thrombosed lesion.^5,11)^ In this case, the reverse slider technique^18)^ was used, which allowed the flow divider of the main body to be deployed in the non-thrombosed segment. In addition, the stent-graft legs were deployed in the thrombosed area and did not expand sufficiently, thus requiring an additional inner-lining stent placement. Third, the prevention of distal embolization may occasionally be necessary. Distal embolization has been reported as a postoperative complication of EVAR.^21)^ In this case, the lesion was considered a chronic occlusion with organized thrombus; therefore, the risk of distal embolization was not considered high, and no other specific embolic protection was used. Although distal embolization did not occur in this case, it may occur during EVAR for thrombosed AAAs, and preventive measures such as femoral artery clamping should be considered, when necessary.^4,5,10,11,13,15)^

## CONCLUSIONS

We reported a case of elective EVAR using a bifurcated endograft for a chronically thrombosed AAA. Although EVAR for a thrombosed AAA is generally considered challenging and requires complex planning and strategies, it can be performed by applying the techniques used in the treatment of LEAD.
